# Modelling dysfunction-specific interventions for seizure termination in epilepsy

**DOI:** 10.1038/s41540-025-00632-9

**Published:** 2025-12-19

**Authors:** Aravind Kumar Kamaraj, Matthew Parker Szuromi

**Affiliations:** 1 Surrey Sleep Research Centre, School of Biosciences, Faculty of Health and Medical Sciences, University of Surrey, Guildford, UK; 2 UK Dementia Research Institute Care Research and Technology Centre at Imperial College, London, and the University of Surrey, Guildford, UK; 3 Graduate Program for Neuroscience, Boston University, Boston, MA USA

**Keywords:** Neurology, Neuroscience

## Abstract

Epileptic seizures result from abnormal synchronous neuronal firing caused by an imbalance between excitatory and inhibitory neurotransmission. While most seizures are self-limiting, those lasting over five minutes, termed status epilepticus, require medical intervention. Benzodiazepines, the first-line treatment, terminate seizures by enhancing GABAergic inhibition, but fail in approximately 36% of cases. In this paper, we employ a neural mass framework to investigate how different interventions influence brain dynamics and facilitate seizure termination. As seizures are characterized by persistent firing, we extend the classic Wilson-Cowan framework by introducing a term called sustenance which encodes factors that promote or discourage perpetual firing. The resulting model captures transitions between normal activity and seizure and provides a tractable framework for analysing diverse pathophysiological mechanisms. We first show how various dysfunctions—such as hyperexcitation, depletion of inhibitory neurotransmitters, and depolarizing GABAergic transmission—can all give rise to seizures, with overlapping but distinct dynamics. Building on this foundation, we turn to the central question of intervention: how different treatments act on these mechanisms to terminate seizures. We find that while enhancing GABAergic inhibition is generally effective, it fails when GABA becomes depolarizing. In such cases, interventions like levetiracetam that suppress sustained excitatory activity remain effective. These findings highlight the importance of aligning interventions to the specific underlying dysfunction for effective seizure termination.

## Introduction

Epilepsy is a neurological disorder characterized by recurrent, unprovoked seizures caused by abnormal brain activity. Seizures reflect episodes of excessive, synchronous neuronal activity in the brain that can lead to transient disruptions in behaviour, sensation, or consciousness. Affecting over 50 million people globally, epilepsy is among the most common neurological conditions^1^. Disruptions in the balance between excitatory and inhibitory neurotransmission play a key role in its pathophysiology; however, the precise mechanisms by which epileptic seizures begin and end are not well understood^2^. Experimental studies have demonstrated that both enhanced excitation^3,4^ and impaired inhibition^5–7^ can lead to seizures. Further, as seizure onset, prediction, and prevention carry greater immediate clinical relevance, they have received considerable research attention, whereas the mechanisms underlying seizure termination remain comparatively under-explored^8^.

Medications are required to terminate a seizure when it becomes prolonged or fails to resolve spontaneously. While most seizures are self-limiting, those lasting over five minutes, termed *status epilepticus*, pose a high risk of neuronal injury, systemic complications, and increased mortality^9^. Benzodiazepines are the first-line treatment due to their rapid onset and potent anticonvulsant effects^10^. They terminate seizures by enhancing GABA-mediated inhibition^11^. Gamma-aminobutyric acid (GABA), the primary inhibitory neurotransmitter in the mature central nervous system, exerts its effects primarily through GABA_*A*_ receptors on neurons^12^. Activation of these receptors opens chloride channels, resulting in chloride influx and neuronal hyperpolarization, thereby reducing neuronal excitability^13^.

However, approximately 36% of status epilepticus cases are refractory to benzodiazepine treatment^14^. In such cases, second-line treatments such as levetiracetam, phenytoin, and sodium valproate are recommended^15^. Most of these agents selectively antagonize rhythmic firing caused by excessive excitatory feedback, while sparing normal electrophysiological function^16^. For instance, phenytoin obstructs pathological excitatory feedback through a voltage-dependent blockade of sodium channels responsible for action potential generation^17^, whereas levetiracetam works by binding to the SV2A ligand and suppressing the release of the excitatory neurotransmitter glutamate^18,19^.

Given that first-line treatments enhance inhibition while second-line treatments suppress excessive excitation, we hypothesize that aligning the therapeutic strategy with the underlying dysfunction could lead to more effective seizure control. To investigate this, we employ a neural mass model to study how different dysfunctions and pharmacological interventions shape seizure dynamics, offering insights into dysfunction-specific treatment approaches.

Mathematical modelling of epilepsy is a broad field, with models ranging from detailed single-neuron representations to large-scale networks encompassing multiple interconnected brain regions^20^. Neural mass models, situated at the mesoscopic scale, provide a useful level of abstraction for studying the dynamics of a small number of interacting neuronal populations^21,22^. Earlier models primarily focussed on replicating electroencephalographic (EEG) features during seizures, normal activity, and the transition between the two states^23,24^. Extensions, such as incorporating distinct fast and slow inhibitory feedback loops, enable the simulation of high-frequency oscillations observed in intracranial EEG recordings^25^ while neural field models incorporating a network of neural masses allow for detailed spatio-temporal description of seizure propagation and termination^26,27^.

Additionally, neural mass models have been tailored to study specific epileptic syndromes by including multiple populations, such as thalamic and cortical populations for absence seizures^28^, and excitatory subpopulations incorporating depolarizing GABAergic neurotransmission for Dravet’s syndrome^29^. However, as these models evolved to capture increasingly complex dynamics, they have often sacrificed clear physiological interpretation for sophistication, making it challenging to directly map the model parameters and insights to underlying mechanisms.

In this paper, we build upon the original Wilson-Cowan model^30^, focusing on a single excitatory and inhibitory population. To capture the defining feature of seizures—persistent neuronal firing—we introduce a term called sustenance, which quantifies factors that either promote or inhibit sustained activity. This term allows us to model both dysfunctions that facilitate seizure-like dynamics and interventions that counteract them. We define the model’s attractors in physiologically grounded terms: the attractor with maximal excitatory activity corresponds to a seizure, and the attractor with minimal, non-zero neuronal activity represents normal brain function. While our abstraction does not capture detailed EEG features, it provides an elegant and tractable framework for examining different seizure mechanisms.

Within this framework, we first investigate how various dysfunctions, such as hyperexcitation, depletion of inhibitory neurotransmitters, and depolarizing GABAergic neurotransmission, can lead to seizure onset. We then turn to the central question of intervention and explore how different treatment strategies act on these mechanisms to terminate seizures.

## Results

A defining feature of epileptic seizures is persistent neuronal firing. When a large fraction of excitatory neurons become active, this heightened activity can sustain pathological firing through positive feedback^31^. To model this mechanism, we start with the Wilson-Cowan model (Eqs. (1 and 2)) and introduce a second-order decay term called sustenance. The level of sustenance within the excitatory and inhibitory populations is quantified by the parameters *q*_*E*_ ∈ [0, 1] and *q*_*I*_ ∈ [0, 1], respectively. The dynamical landscape of the resulting model is defined by two first-order ordinary differential equations: Eq. (7) governing excitatory activity, *E*, and Eq. (8) governing inhibitory activity, *I*. Here, the term ‘activity’ refers to the proportion of neurons firing at any given time *t*. Note that time *t* is a dimensionless quantity in our analyses.

**Fig. 1:**
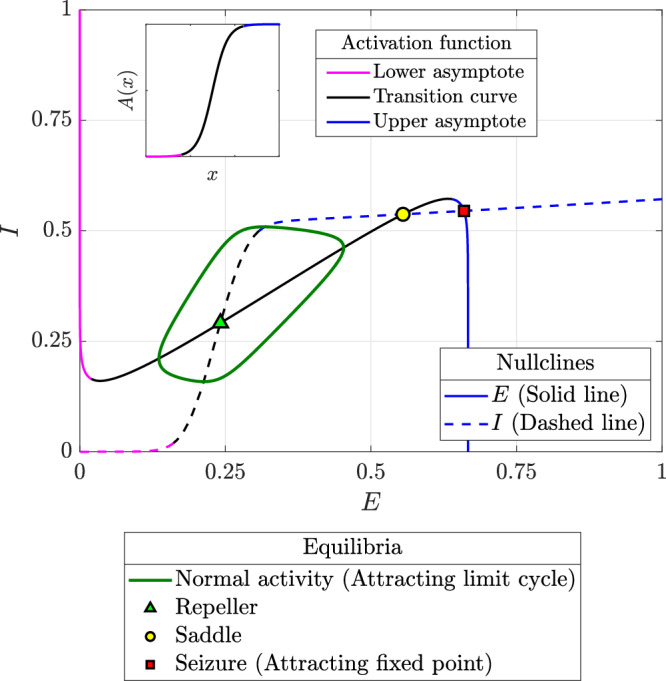
Illustrative phase portrait showing the *E*- and *I*-nullclines, with the different segments of the nullclines coloured to correspond with the respective segments of the activation function (inset). The different equilibria and limit cycles are also indicated therein: two attractors, one corresponding to normal activity and the other to seizure, a repeller, and a saddle.

This two-dimensional framework lends itself to a visual and intuitive understanding of brain dynamics, as perturbations to excitatory or inhibitory mechanisms manifest directly as geometric changes in the *E*- and *I*-nullclines (Eqs. (9 and 10), respectively). Accordingly, we use phase portraits such as the one shown in Fig. 1, to illustrate these changes and extract qualitative insights.

The geometry of the nullclines is shaped by the sigmoidal activation functions (Eqs. (5 and 6)), each of which can be divided into three distinct regions: the upper asymptote (*A*_*E*_, *A*_*I*_ → 1), the lower asymptote (*A*_*E*_, *A*_*I*_ → 0), and the transition curve (the remaining segment in the middle) as shown in the inset in Fig. 1. These three regions correspond to distinct segments of the *E*- and *I*-nullclines, which we use to interpret the model’s dynamics. We begin our analysis by identifying the different equilibria and limit cycles that emerge at the intersections between these nullcline segments: normal activity, seizure, and saddles.

We define normal activity as the attractor characterized by minimal but non-zero excitatory and inhibitory activity. This attractor arises at the first intersection of the segments of the *E-* and *I*-nullclines that correspond to the transition regions (plotted in black in Fig. 1) of their respective activation functions. The transition segments ensure non-zero activity, while the first intersection guarantees this activity remains minimal. Depending on system dynamics, normal activity can appear as either an attracting fixed point at this intersection or, if the fixed point is repelling, an attracting limit cycle centred around it. The latter case is illustrated in Fig. 1, where the limit cycle is shown in dark green and the repelling fixed point as a pale green triangle.

A seizure in our framework corresponds to the attractor formed at the intersection of upper asymptote segments of the *E*- and *I*-nullclines. As with normal activity, seizure can appear as either an attracting fixed point at this intersection or, if the fixed point is repelling, an attracting limit cycle centred around it. The former case is illustrated in Fig. 1, where the seizure fixed point is shown as a red square. Since the upper asymptote represents maximal activation, the seizure attractor reflects a state of runaway excitation that inhibitory mechanisms, even at full activation, cannot control.

The equilibria formed at other intersections between the *E*- and *I*-nullclines are either repellers or saddles, forming barriers that separate the two attractors of interest: normal activity and seizure.

### Baseline model

To serve as the reference point for our analyses, we establish a ‘baseline model’ with parameters configured such that a limit cycle representing normal activity is the only attractor in the system. The specific parameter values for this baseline model are listed in Table 1, and the corresponding phase portrait is shown in Fig. 3a.

**Table 1:** Parameters for the baseline model

Connection weights				Activation function			
*a*_*EE*_	*a*_*EI*_	*a*_*IE*_	*a*_*II*_	*θ*_*E*_	*μ*_*E*_	*θ*_*I*_	*μ*_*I*_
10	10	12	1	3	1.5	5	2.7

Rate constants			External drive		Sustenance		
*τ*_*E*_	*τ*_*I*_		*D*_*E*_	*D*_*I*_	*q*_*E*_	*q*_*I*_	
1	1		0.25	0	0.75	0.25	

In the following sections, we examine how different excitatory and inhibitory dysfunctions modify the geometry of the nullclines, thereby shaping brain dynamics and triggering seizure onset. For each dysfunction, we begin from the baseline and progressively vary a control parameter to demonstrate how increasing dysfunction transitions the system from a single attractor corresponding to normal activity, to a bistable regime with coexisting normal and seizure states, and ultimately to seizure as the only attractor.

### Seizures arising from hyperexcitation

We model hyperexcitation as an increase in the net drive to the excitatory population (*D*_*E*_) and analyse the conditions under which it can trigger a seizure.

**Fig. 2:**
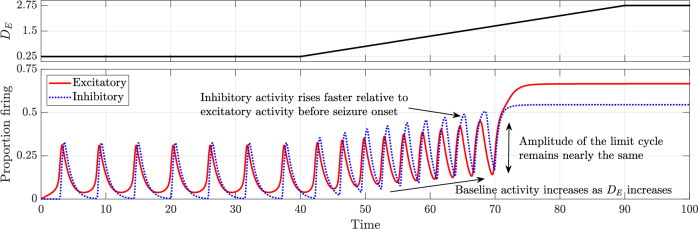
Time series depicting the transition from normal activity to seizure as the drive to the excitatory population *D*_*E*_ is increased linearly. All other parameters remain as in the baseline model.

Figure 2 illustrates the evolution of neuronal activity over time as the control parameter *D*_*E*_ increases linearly. All parameters remain fixed at baseline values except for *D*_*E*_, which is initially held at 0.25 between *t* = 0 and *t* = 40, where the system exhibits a limit cycle corresponding to normal activity. From *t* = 40 to *t* = 90, *D*_*E*_ increases linearly from 0.25 to 2.75 and is then maintained at 2.75.

**Fig. 3:**
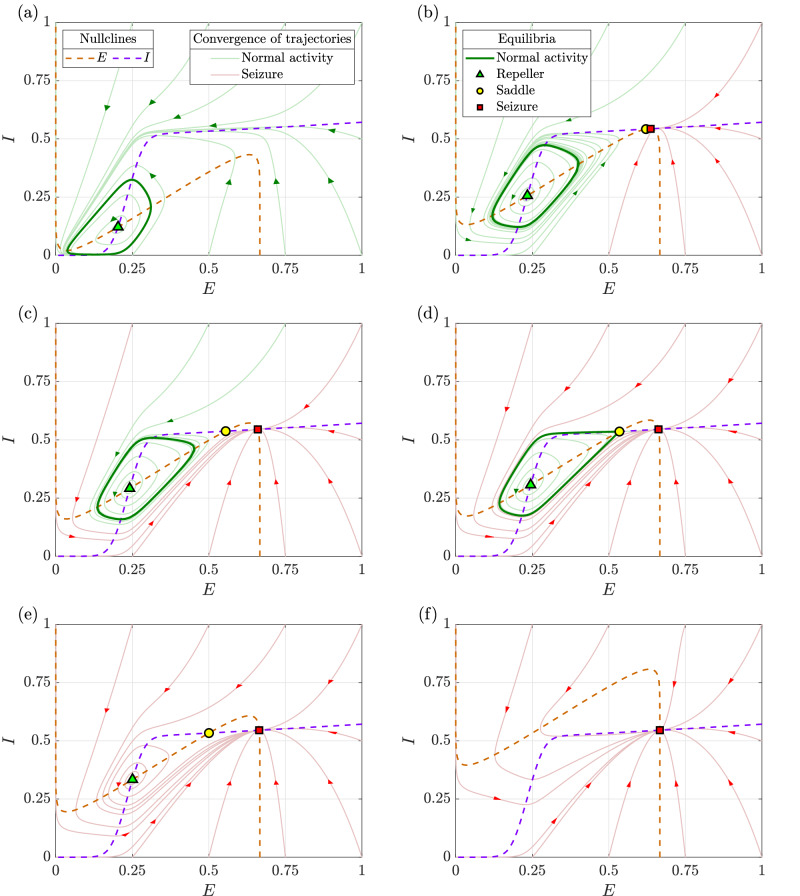
Phase portraits illustrating the change in dynamics with an increase in the drive to the excitatory population (*D*_*E*_). **a** *D*_*E*_ = 0.25: normal activity is the sole attractor, **b** *D*_*E*_ = 1.36: birth of seizure attractor and saddle through a saddle-node bifurcation, **c** *D*_*E*_ = 1.65: bistability between normal activity and seizure, **d** *D*_*E*_ = 1.7751: normal activity vanishes through a saddle-homoclinic bifurcation, **e** *D*_*E*_ = 2: seizure is the sole attractor, while the saddle and repeller persist as non-attracting equilibria, and **f** *D*_*E*_ = 4: seizure remains the sole equilibrium. All other parameters remain as in the baseline model.

**Fig. 4:**
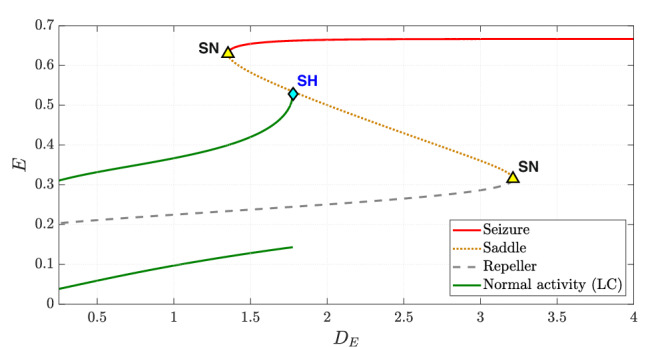
Bifurcation diagram illustrating the change in dynamics with an increase in the drive to the excitatory population (*D*_*E*_). All other parameters remain as in the baseline model. The two solid green lines indicate the extrema of the limit cycle describing normal activity while the solid red line represents seizure. The saddle-node bifurcation is marked as “SN” and the saddle-homoclinic bifurcation is marked as “SH”.

As *D*_*E*_ increases, the limit cycle retains a nearly constant amplitude while baseline activity steadily rises. Inhibitory activity rises sharply relative to excitatory activity, reflecting inhibition’s effort to counteract the growing excitation. The transition from normal activity to seizure occurs precisely when the limit cycle describing normal activity vanishes through the saddle-homoclinic bifurcation. At the bifurcation point of *D*_*E*_ ≈ 1.7751, excitatory activity jumps suddenly, marking seizure onset. Beyond this threshold, the system remains in the seizure attractor. This transition is further illustrated through phase portraits in Fig. 3 and the bifurcation diagram in Fig. 4.

Figure 3a illustrates the phase portrait with a low excitatory drive (*D*_*E*_ = 0.25). Here, the *E-* and *I*-nullclines intersect only once and the resulting fixed point (green triangle) is repelling. A stable limit cycle (solid green curve) surrounds this point, representing normal activity. Since all trajectories converge onto this limit cycle, it is globally attracting.

Increasing *D*_*E*_ shifts the *E*-nullcline away from the *E*-axis, while the *I*-nullcline remains unchanged. At *D*_*E*_ ≈ 1.353, a second intersection between the two nullclines occurs, leading to a saddle-node bifurcation. As shown in the bifurcation diagram in Fig. 4, this bifurcation creates two new fixed points: a saddle and an attractor, the latter corresponding to seizure. Figure 3b depicts the phase portrait just after this bifurcation (*D*_*E*_ = 1.36), where the emergence of the seizure attractor (red square) introduces bistability. The trajectories converging to normal activity and seizure are shown in green and red, respectively. Notably, the time series does not immediately reflect this transition from monostability to bistability.

As *D*_*E*_ continues to increase, the limit cycle representing normal activity gradually moves closer to the saddle, as shown in Fig. 3c. At *D*_*E*_ ≈ 1.7751, the limit cycle collides with the saddle and disappears through a saddle-homoclinic bifurcation as shown in Fig. 4. Figure 3d depicts the phase portrait corresponding to this bifurcation point, where all trajectories originating inside the limit cycle still approach it, while those originating outside it converge onto the seizure attractor.

Further increasing *D*_*E*_ destroys bistability, making seizure the global attractor. Figure 3e illustrates this scenario with *D*_*E*_ = 2, where the repeller and the saddle remain as non-attracting equilibria. These two equilibria persist until *D*_*E*_ ≈ 3.4236, at which point they annihilate each other in a saddle-node bifurcation as shown in Fig. 4. Beyond this threshold, seizure becomes the sole equilibrium state, as depicted in Fig. 3f.

### Seizures arising from the depletion of inhibitory neurotransmitter

Seizures can also arise from dysfunctions in inhibition. For example, when inhibitory neurons fire at high frequencies for prolonged periods, the mechanisms that sustain synaptic function, such as neurotransmitter production and vesicle recycling, may fail to keep pace with demand^32^. This can lead to depletion of inhibitory neurotransmitters like GABA at excitatory postsynaptic neurons, thereby weakening inhibitory feedback and increasing susceptibility to seizures^33,34^. We model the propensity for inhibitory neurotransmitter depletion using Eq. (13), which describes the effective inhibition felt by postsynaptic neurons. The level of depletion is quantified by the parameter *ρ* ∈ [0, 1], where *ρ* = 0 indicates no depletion and *ρ* = 1 indicates maximum depletion.

**Fig. 5:**
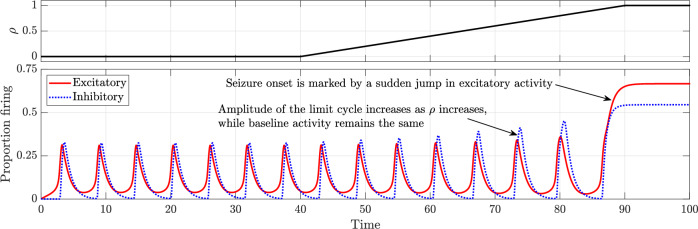
Time series depicting the transition from normal activity to seizure as the depletion parameter *ρ* is increased linearly. All other parameters remain as in the baseline model.

**Fig. 6:**
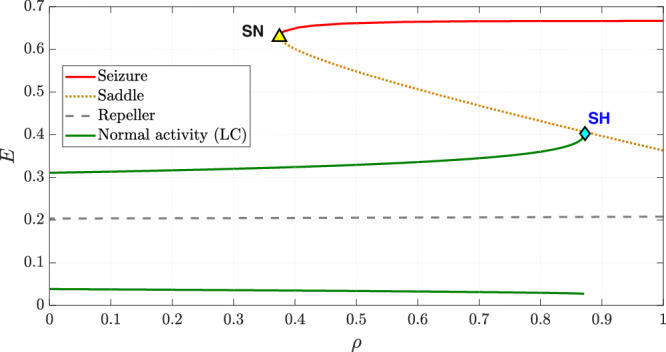
Bifurcation diagram illustrating the change in dynamics with an increase in the depletion parameter *ρ.* All other parameters remain as in the baseline model. The two solid green lines indicate the extrema of the limit cycle describing normal activity while the solid red line represents seizure. The saddle-node bifurcation is marked as “SN” and the saddle-homoclinic bifurcation is marked as “SH”.

Figure 5 illustrates the evolution of neuronal activity as the depletion parameter *ρ* increases linearly with time. The bifurcation diagram depicting the evolution of dynamics with *ρ* is shown in Fig. 6. All parameters, except *ρ*, are set as in the baseline model and held constant.

At *ρ* = 0, the system exhibits a globally attracting limit cycle corresponding to normal activity. As *ρ* increases from *t* = 40, the time series in Fig. 5 shows that the limit cycle slowly grows in amplitude, while the baseline activity remains unchanged. This contrasts with the case of increased drive to the excitatory population, where baseline activity rises while the amplitude of the limit cycle remains constant.

The bifurcation diagram in Fig. 6 shows that, at *ρ* ≈ 0.3744, a saddle-node bifurcation occurs, giving rise to a saddle and the seizure attractor leading to bistability. As with the hyperexcitation case, the transition from monostability to bistability is not immediately apparent in the time series.

As *ρ* increases further, the limit cycle continues to expand until it eventually collides with the saddle and disappears through a saddle-homoclinic bifurcation at *ρ* ≈ 0.874 as shown in Fig. 6. This destroys bistability, leaving seizure as the sole attractor. In the time series, this saddle-homoclinic bifurcation corresponds to seizure onset, characterized by a sudden jump in excitatory activity as the trajectory transitions from the limit cycle to the seizure attractor.

Phase portraits depicting key phases of this transition are provided in Supplementary Fig. S1.

### Seizures arising from the depolarising effect of GABAergic neurotransmission

The inhibitory neurotransmitter gamma-aminobutyric acid (GABA) exerts its effects predominantly through the activation of GABA_*A*_ receptors on neurons. Under normal conditions, activation of these receptors opens chloride channels, resulting in chloride influx and neuronal hyperpolarization, provided that the chloride equilibrium potential is more negative than the resting membrane potential^13^. However, in developmental stages or pathological conditions such as epilepsy, alterations in chloride transporter expression can disrupt this balance. Specifically, an imbalance between chloride influx via the sodium-potassium-chloride co-transporter (NKCC1) and efflux via the potassium-chloride co-transporter (KCC2) can lead to persistent intracellular chloride accumulation^35^. We model this transporter imbalance using the parameter *κ*, where *κ* = 0 represents intact homeostasis and positive values indicate impairment, with higher values reflecting greater dysfunction.

When intracellular chloride levels become sufficiently elevated, the chloride reversal potential shifts toward more depolarized values, causing a switch in GABA_*A*_ receptor activation from chloride influx to efflux. This reversal of GABA’s effect from inhibition to excitation undermines inhibitory restraint and can promote seizures^36,37^. To capture this phenomenon, we subdivide the quiescent excitatory population into two subpopulations, the subpopulation *p* where GABA becomes excitatory (Eq. (14)) and the remaining subpopulation 1 − *p* where GABA remains inhibitory (Eq. (3)). We model the resultant perception felt by the quiescent excitatory population by combining the two subpopulations using Eq. (15).

**Fig. 7:**
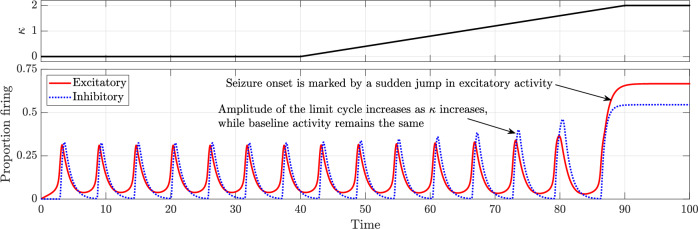
Time series depicting the transition from normal activity to seizure as the chloride accumulation parameter *κ* is increased linearly. All other parameters remain as in the baseline model.

**Fig. 8:**
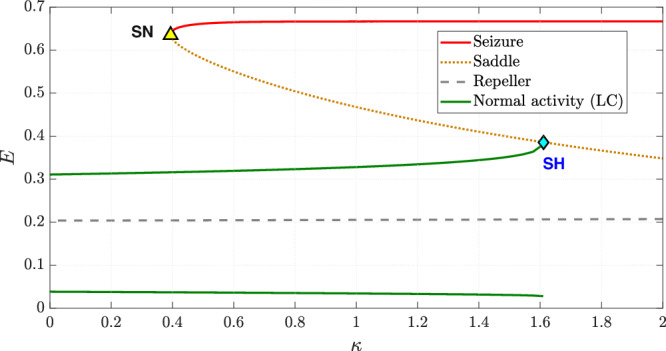
Bifurcation diagram illustrating the change in dynamics with an increase in the chloride accumulation parameter *κ.* All other parameters remain as in the baseline model. The two solid green lines indicate the extrema of the limit cycle describing normal activity while the solid red line represents seizure. The saddle-node bifurcation is marked as “SN” and the saddle-homoclinic bifurcation is marked as “SH”.

We then explore the evolution of neuronal activity by gradually increasing the chloride accumulation parameter *κ* over time. The sensitivity parameter in Eq. (14), quantifying the effect of depolarising GABA on postsynaptic neurons, *a*_*PI*_, is held constant at 5. All other parameters are set as in the baseline model and held constant. The results, shown in Figs. 7 and 8, closely resemble those observed in the case of inhibitory neurotransmitter depletion, with the transition from normal activity to seizure occurring via a saddle-homoclinic bifurcation at *κ* ≈ 1.61714.

Phase portraits depicting key phases of this transition are provided in Supplementary Fig. S2.

### Interventions for seizure termination are influenced by the underlying dysfunction

Having analysed how various dysfunctions contribute to seizure onset, we now turn to their implications for interventions aimed at seizure termination. In our model, seizure termination is defined as the disappearance of the seizure attractor via a saddle-node bifurcation, rather than simply restoring bistability. As noise has not been included, the system remains in the seizure attractor until it vanishes through a saddle-node bifurcation, provided the system is initialized in the seizure state. If noise were present, it could drive transitions both into and out of the seizure attractor within the bistable regime; thus, eliminating the seizure attractor is also important to prevent immediate recurrence.

Since benzodiazepines, the first-line treatment, terminate seizures by enhancing GABAergic inhibition, we begin by investigating the effects of GABAergic enhancement on neuronal dynamics within our model. We represent GABAergic enhancement by introducing a factor, *σ*_GABA_, which amplifies the effect of inhibition in the argument of the excitatory activation function as described in Eq. (16). To illustrate this, we first consider seizures driven by hyperexcitation. All parameters are set as in the baseline model, except for the drive to the excitatory population, which is set to *D*_*E*_ = 3 to ensure seizure remains the sole attractor.

**Fig. 9:**
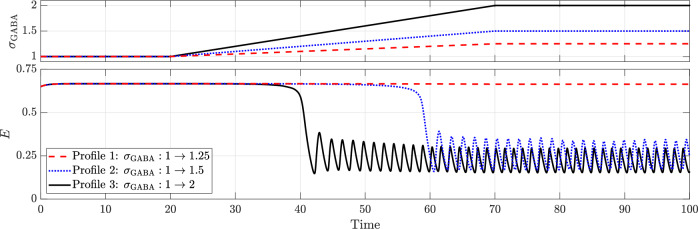
Effect of GABAergic enhancement in terminating seizures caused due to hyperexcitation (*D*_*E*_ = 3). All other parameters remain as in the baseline model.

Figure 9 illustrates the effect of three levels of GABAergic enhancement on excitatory activity (*E*). Inhibitory activity is omitted for clarity, as excitatory activity alone is sufficient to determine whether the brain is in a seizure or normal state. Between *t* = 0 and *t* = 20, GABAergic enhancement is held at the baseline level of *σ*_GABA_ = 1 and the system remains in a seizure state. Between *t* = 20 and *t* = 70, *σ*_GABA_ is increased linearly to three different levels, 1.25, 1.5 and 2, and held at these levels thereafter.

When *σ*_GABA_ peaks at 1.25, seizure termination is unsuccessful. However, higher levels (*σ*_GABA_ = 1.5 and 2) successfully terminate the seizure, restoring normal activity characterized by a limit cycle. The limit cycle does not recover the same amplitude as before the epileptic dysfunction. Instead, it settles at a different amplitude that depends on the level of intervention applied. Additionally, comparing *σ*_GABA_ = 1.5 and 2 reveals that stronger GABAergic enhancement results in faster seizure termination and a lower proportion of neurons firing during normal activity. The bifurcation diagram and phase portraits depicting key phases of this transition are provided in Supplementary Figs. S3 and S4, respectively.

**Fig. 10:**
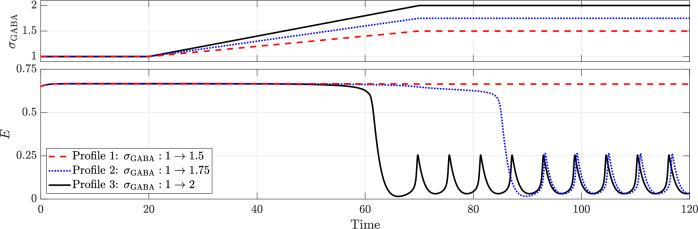
Effect of GABAergic enhancement in terminating seizures caused due to the depletion of inhibitory neurotransmitter (*ρ* = 1). All other parameters remain as in the baseline model.

A similar effect is observed when GABAergic enhancement is employed to terminate seizures caused by depletion of inhibitory neurotransmitter, as shown in Fig. 10, but with two key differences. First, numerical bifurcation analysis indicates that successful termination requires higher levels of GABAergic enhancement in the depletion case (*σ*_GABA_ ≈ 1.74285) compared to seizures driven by hyperexcitation (*σ*_GABA_ ≈ 1.3035). Second, in the depletion case, the proportion of excitatory neurons firing during normal activity is perturbed to a smaller extent than in the hyperexcitation case. This arises because the inhibition scales as *σ*_GABA_*I* in the hyperexcitation case, whereas in the depletion case it scales as *σ*_GABA_*I*^eff^, with *I*^eff^ < *I*. Consequently, a higher level of GABAergic enhancement is required for termination, and even then, the inhibitory restraint is insufficient to markedly reduce the proportion of excitatory neurons firing. For completeness, the corresponding bifurcation diagram and phase portraits are provided in Supplementary Figs. S5 and S6, respectively.

**Fig. 11:**
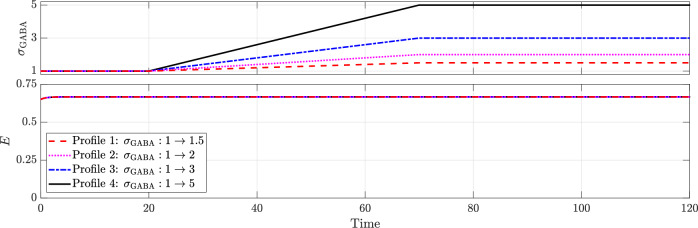
GABAergic enhancement fails to terminate seizures when GABAergic neurotransmission is depolarising (*κ* = 1.8 and *a*_*PI*_ = 5). All other parameters remain as in the baseline model.

On the other hand, when GABAergic neurotransmission becomes depolarising, GABAergic enhancement fails to terminate the seizure, as shown in Fig. 11. This failure occurs because GABA’s role switches from inhibitory to excitatory, so enhancing its effect no longer suppresses seizure activity.

To terminate such benzodiazepine-refractory seizures, second-line treatments such as levetiracetam and phenytoin, which selectively antagonize rhythmic firing, are recommended^15^. In our framework, the sustenance terms, *q*_*E*_ and *q*_*I*_, encode factors responsible for persistent firing through a second-order decay. Consequently, to model the selective suppression of rhythmic activity, we introduce a term *σ*_RS_ that counteracts sustenance by modifying Eqs. (7 and 8), replacing *q*_*E*_ and *q*_*I*_ with (*q*_*E*_ − *σ*_RS_) and (*q*_*I*_ − *σ*_RS_), respectively. Note that the term ‘rhythmic suppression’ for *σ*_*RS*_ refers specifically to the suppression of abnormal rhythmic discharges observed in brain recordings during seizures under the action of certain medications, and is not intended to describe the limit cycles that arise in the model dynamics.

**Fig. 12:**
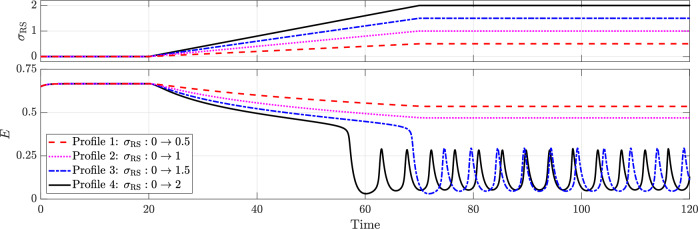
Effect of rhythmic suppression (*σ*_RS_) in terminating seizures driven by depolarising GABAergic neurotransmission (*κ* = 1.8 and *a*_*PI*_ = 5). All other parameters remain as in the baseline model.

Figure 12 illustrates the effect of varying levels of rhythmic suppression on excitatory activity (*E*). From *t* = 0 to *t* = 20, there is no rhythmic suppression (*σ*_RS_ = 0), and the system remains in a seizure state. Between *t* = 20 and *t* = 70, *σ*_RS_ is increased linearly to four different levels, 0.5, 1, 1.5, and 2, after which it is held constant.

Seizure termination occurs at *σ*_RS_ ≈ 1.35375, as confirmed by the bifurcation diagram provided in the supplementary material (Fig. S7). While lower levels of rhythmic suppression do not fully stop the seizure, they still reduce neuronal activity during seizures—an effect not observed with GABAergic enhancement. Increasing *σ*_RS_ results in a greater suppression of activity; for levels sufficient to achieve termination, higher doses lead to faster termination as well as a slight increase in the frequency of the limit cycle representing normal activity, as shown in Fig. 12. The bifurcation diagram and phase portraits depicting key phases of this transition are provided in Supplementary Figs. S7 and S8, respectively.

**Fig. 13:**
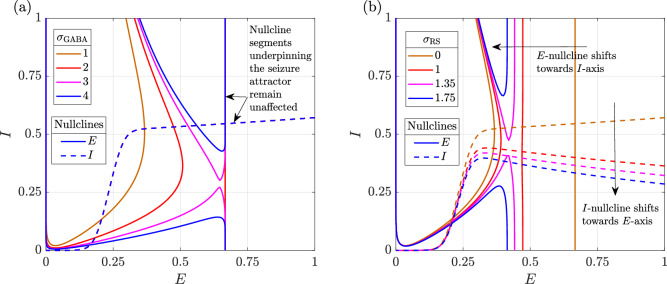
Influence of various interventions on the *E*- and *I*-nullclines when GABAergic neurotransmission is depolarising (κ = 1.8 and a_PI_ = 5). **a** GABAergic enhancement and **b** rhythmic suppression. All other parameters remain as in the baseline model.

To understand why rhythmic suppression can terminate seizures when GABAergic enhancement fails, we compare their effects on the *E*- and *I*-nullclines under conditions of depolarizing GABAergic neurotransmission (Fig. 13). Rhythmic suppression, by counteracting the sustenance term, shifts the upper asymptote segments of both nullclines that underpin the seizure attractor and alters the position of the seizure attractor in phase space. Specifically, it moves the *E*-nullcline toward the *I*-axis and the *I*-nullcline toward the *E*-axis, thereby reducing the level of activity during seizure. This progressive shift ultimately leads to the collision and mutual annihilation of the seizure attractor and the adjacent saddle point, resulting in seizure termination. In contrast, GABAergic enhancement via benzodiazepines affects only the excitatory activation function and does not shift the seizure attractor. Consequently, it neither reduces neuronal activity during seizures nor achieves termination. These findings underscore the importance of aligning therapeutic strategies with the specific pathophysiological mechanisms for effective seizure termination.

## Discussion

We now turn to a discussion of the key features of our model, its limitations, and potential avenues for future extensions. The goal of this paper is to demonstrate, through a phenomenological model, that the optimal choice of treatment for seizure termination depends on the underlying dysfunction.

A defining feature of epileptic seizures is persistent neuronal firing, which can arise from several biophysical processes. For instance, the activation of N-methyl-D-aspartate (NMDA) receptors is known to drive persistent firing of excitatory cells and is dependent upon sufficient depolarization^38^. Changes in NMDA receptor abundance or subunit composition may further amplify this effect^39,40^. Abnormal recurrent excitatory connectivity can likewise reinforce pathological activity patterns^31^.

Previous modelling studies have incorporated amplification or suppression of activity under elevated excitation by modifying the activation function, whereas in our approach, this effect is captured by altering the decay term while leaving the activation function unchanged.

For instance, introducing superlinearity into the activation function by adding a quadratic term to its input creates bistability in a stochastic Wilson-Cowan framework. The resulting coexisting attractors correspond to low-activity states resembling normal brain activity and high-activity states resembling seizure dynamics^41^. In another study, a complex neural-mass framework is employed in which multiple plasticity processes are incorporated, including NMDA/Ca^2+^-mediated potentiation, AMPA receptor insertion, chloride-handling changes via KCC2, and extra-synaptic GABAergic degradation. Together, these mechanisms progressively reshape the sigmoidal activation function by altering its slope and threshold, embedding positive feedback that enlarges the seizure-prone regime and enables secondary epileptic foci^42^. In another approach, a neural-mass model of cortical circuits was used in which an internal feedback controller was introduced to regulate excitatory coupling. When the controller was impaired, the activation function was effectively steepened, resulting in hypersynchronous seizure-like oscillations, whereas the application of external feedback restored the activation function toward its baseline form and suppressed pathological activity^43^. Suppression of activity at high excitatory input can be modelled by replacing the sigmoidal activation function with a Gaussian^44^. However, accurately reproducing partial suppression rather than complete silencing requires a finely tuned combination of a Gaussian and a sigmoid, which increases model complexity and reduces interpretability.

**Fig. 14:**
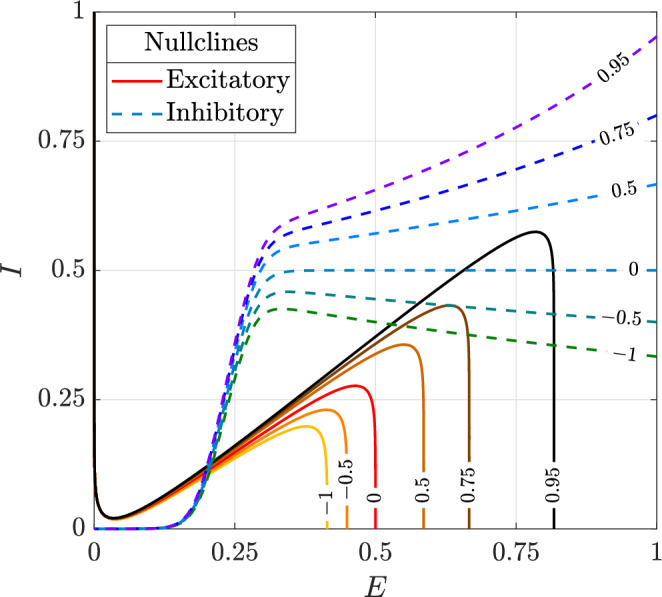
Influence of the second-order decay term on the excitatory (solid lines) and inhibitory (dashed lines) nullclines. Each plot is labelled with the coefficient of the net second-order decay, defined as the contribution from sustenance minus that from rhythmic suppression. In our model, the coefficients for excitatory and inhibitory populations can be varied independently, affecting only the *E*- and *I*-nullclines, respectively. The two nullclines are shown together to illustrate all possible combinations.

In contrast, our model captures persistent activity and its modulation through a second-order decay, striking a balance between model fidelity and complexity. Persistent firing is represented by a positive second-order decay term (sustenance), while the therapeutic effect of rhythmic suppression is modelled as a negative second-order decay term. This second-order decay primarily influences the upper asymptotic segments of the nullclines: shifting the position of the *E*-nullcline and altering the slope of the *I*-nullcline (Fig. 14). Consequently, if the net second-order effect (sustenance minus rhythmic suppression) is positive, firing rates during seizure increase, whereas if it is negative, firing rates decrease. By contrast, normal activity remains largely unaffected, as the lower asymptotic and transition segments are only minimally perturbed by the second-order decay term (Fig. 14). This behaviour is consistent with the action of second-line seizure treatments such as phenytoin and levetiracetam: although these drugs act through distinct mechanisms, both suppress abnormal firing in epileptic circuits while sparing normal electrophysiological function^45,46^. Our model phenomenologically reproduces this selectivity, and the results support their efficacy as alternatives for benzodiazepine-refractory seizures, in line with clinical observations^47,48^.

For the sustenance parameters, a relatively high excitatory sustenance (*q*_*E*_) was chosen to encode the model’s increased propensity to generate seizures. Inhibitory sustenance was set lower, consistent with our restriction of sustenance values to the range [0, 1]. A negative sustenance could alternatively be used to represent depolarization block, in which elevated excitation paradoxically produces reduced firing rates, as demonstrated in ref. ^44^.

In the original Wilson-Cowan model^30^, normal activity is typically represented by the trivial fixed point (0, 0). In contrast, normal activity in our framework is non-trivial, better reflecting physiological observations that a small but non-zero fraction of excitatory and inhibitory neurons remain active in a healthy brain. Furthermore, the seizure attractor corresponds to uncontrolled excitation that persists despite maximal inhibitory activation. While earlier studies on neuronal ensembles have explored non-trivial, periodic solutions to represent normal activity^49,50^, our work offers a formal definition within the Wilson-Cowan framework: normal activity is defined as the stable attractor arising from the first intersection of the transition segments of the *E*- and *I*-nullclines, whereas, seizure corresponds to the stable attractor formed by the intersection of the upper asymptotic segments. This formalism enables a physiologically interpretable classification of the dynamic regimes in epilepsy.

Seizures are thought to arise from transient, activity-dependent imbalances between excitation and inhibition^51^. To reflect this, we have included an activity-dependent contribution whilst modelling dysfunctions. Specifically, the model for inhibitory neurotransmitter depletion assumes that the level of depletion is proportional to the average inhibitory activity over a preceding time window. Similarly, the model for depolarising GABAergic neurotransmission reflects the dependence of neuronal depolarization and GABA_*A*_ activation on ongoing excitatory and inhibitory activity, respectively. While modelling hyperexcitation, the increased excitatory drive *D*_*E*_ is not constructed to be explicitly activity-dependent; however, it implicitly captures elevated excitatory input from neighbouring neuronal populations.

Our analyses reveal that the pathological perturbations examined here, whether excitatory or inhibitory, primarily impact the *E*-nullcline. Regardless of the specific dysfunction, as the control parameter varies, the seizure attractor emerges via a saddle-node bifurcation, while normal activity disappears through a saddle-homoclinic bifurcation. The parameter space between these bifurcations corresponds to a bistable regime, where both normal activity and seizure coexist. Although seizure onset occurs at the saddle-homoclinic bifurcation in a noise-free system, real-world neural activity is inherently noisy. Consequently, the transition to seizure can occur at any point after the seizure attractor appears.

Furthermore, our simulations consistently show that, across different dysfunctions, inhibitory activity rises sharply relative to excitation just before seizure onset. The onset itself is then marked by a sudden surge in excitatory activity. This dynamic aligns with physiological observations that inhibitory activity often intensifies prior to seizure onset^52^.

While all dysfunctions primarily affect the *E*-nullcline, the way each one alters its geometry is unique, and these changes are reflected indirectly in the time series through the behaviour of the limit cycle. Specifically, inhibitory dysfunctions cause the amplitude of the limit cycle corresponding to normal activity to increase before seizure onset, whereas hyperexcitation raises baseline activity and increases the frequency of the limit cycle without altering its amplitude.

In terms of interventions, GABAergic enhancement by benzodiazepines primarily influences the geometry of the *E*-nullcline, whereas suppression of rhythmic firing exerts a substantial influence on both the *E*- and *I*-nullclines. When seizures are driven by hyperexcitation or depletion of inhibitory neurotransmitters, shifting the *E*-nullcline alone is sufficient for seizure termination—an effect successfully achieved through GABAergic enhancement. However, when GABAergic neurotransmission becomes depolarizing, enhancing GABAergic inhibition no longer shifts the *E*-nullcline sufficiently to eliminate its intersection with the *I*-nullcline that generates the seizure attractor. In this case, bistability between seizure and normal activity may be restored, but if the system is initialized in the seizure state, it remains trapped there.

In contrast, suppression of rhythmic firing directly perturbs the seizure attractor by reducing the proportion of neurons firing at maximal activation. By shifting the upper asymptote segments of the *E*- and *I*-nullclines, it brings the seizure attractor and the adjacent saddle point together until they collide and annihilate through a saddle-node bifurcation. This eliminates the seizure attractor and restores normal activity.

These findings highlight the need for a systematic approach to treatment selection. While control-theoretic approaches have been explored for optimizing brain stimulation in seizure termination^53,54^, similar systematic studies for drug-based interventions remain limited. Our framework provides a foundation for addressing this gap, enabling the optimization of drug type, dosage, and timing by minimizing a cost function over the parameter space.

In terms of model-data fusion, previous work has demonstrated that features such as EEG amplitude, inter-spike intervals, and the presence of a DC shift can be used to identify patterns that are compatible with different co-dimension one bifurcations at seizure onset and offset^55,56^. In pediatric status epilepticus, scalp EEG was combined with dynamic causal modelling to infer synaptic coupling changes and map them onto a parameter space. Among the parameters considered, alterations in GABAergic synaptic coupling were identified as key indicators of benzodiazepine responsiveness^57^. In a similar way, our model could be extended to serve as a bridge, allowing EEG features to be interpreted through the lens of nullcline geometry and bifurcation structure, thereby linking observed dynamics to underlying dysfunctions and guiding targeted interventions.

Our current model does not account for shunting inhibition, a mechanism by which inhibitory neurons reduce excitability by increasing membrane conductance and diverting electrical currents^58,59^. Shunting inhibition is particularly relevant when GABAergic currents are depolarizing, as the resting membrane potential remains slightly above the chloride reversal potential. In this scenario, even if chloride efflux occurs, the increased conductance allows inhibitory shunting to counteract excitation, preserving the net inhibitory effect of GABA^60^. Future extensions of our model will feature shunting inhibition through a hybrid formulation^61,62^.

Since seizures arise from abnormal synchronous neuronal firing, incorporating synchronization into our model is a natural next step. Recent extensions of the Wilson-Cowan model have integrated synchronization^63^, and similar approaches can enhance our framework. Given that epilepsy is fundamentally a disorder of cortical network disorganization^64^, extending the model to a network of interconnected excitatory and inhibitory populations is essential. Future work will focus on expanding our framework to multiple, interacting populations (for example, pyramidal cells and distinct classes of interneurons) and assessing how insights from the two-population model translate to network-level dynamics.

In summary, our work highlights that the effectiveness of an intervention in epilepsy depends critically on the underlying dysfunction that gives rise to seizures. We show that different pathological mechanisms, namely, hyperexcitation, depletion of inhibitory neurotransmitters, and depolarizing GABA, lead to seizures through distinct routes, and that treatments act by reshaping the dynamical landscape in correspondingly distinct ways. GABAergic enhancement can terminate seizures driven by hyperexcitation or depletion of inhibitory neurotransmitters, but fails when GABA itself becomes depolarising. Rhythmic suppression succeeds under such conditions by countering pathological excitation. These results emphasize that treatment choice cannot be generic: aligning the intervention to the underlying dysfunction is key to effective seizure termination. More broadly, the framework illustrates how phenomenological models can provide an intuitive map between dysfunction, intervention, and outcome, guiding rational treatment selection in epilepsy.

## Methods

We begin with the original Wilson-Cowan equations, which describe the dynamics of interacting excitatory and inhibitory neuronal populations, and progressively incorporate additional features to capture mechanisms relevant to epilepsy. The Wilson-Cowan model is given by ref. ^30^,

1 $$\dot{E}={\tau }_{E}\left({A}_{E}(1-E)-E\right)$$

2 $$\dot{I}={\tau }_{I}\left({A}_{I}(1-I)-I\right)$$

with dynamics restricted to *Ω* = [0, 1] × [0, 1]. The state variables *E* and *I*, respectively, denote the proportion of excitatory and inhibitory neurons engaged in firing at any given time *t*. The parameters *τ*_*E*_ and *τ*_*I*_ denote the rate constants for the excitatory and inhibitory populations, respectively. The activation functions *A*_*E*_ = *f*(*x*_*E*_) and *A*_*I*_ = *g*(*x*_*I*_) govern the recruitment into firing for excitatory and inhibitory populations, respectively. The arguments, *x*_*E*_ and *x*_*I*_, encode how quiescent neurons perceive their input: a weighted sum of excitatory *E* and inhibitory *I* activity, and a net drive,

3 $${x}_{E}={a}_{EE}E-{a}_{EI}I+{D}_{E}$$

4 $${x}_{I}={a}_{IE}E-{a}_{II}I+{D}_{I}$$

where *D*_*E*_ and *D*_*I*_ represent the net drive for the excitatory and inhibitory populations, respectively, and *a*_*ij*_ represent the weight of connection exerted by population *j* on population *i*, with *i*, *j* ∈ {*E*, *I*}.

Given that a higher net excitatory activity can promote recruitment, activation functions are typically considered to be monotonically increasing functions, such as a sigmoid^65^. We choose,

5 $${A}_{E}={\left(1+{e}^{-{\theta }_{E}({x}_{E}-{\mu }_{E})}\right)}^{-1}$$

6 $${A}_{I}={\left(1+{e}^{-{\theta }_{I}({x}_{I}-{\mu }_{I})}\right)}^{-1}.$$

Both activation functions have a lower asymptote at zero and an upper asymptote at one. The parameter *μ*_*i*_ specifies the value of *x*_*i*_ at which the activation function reaches its median value of 0.5, and the parameter *θ*_*i*_ controls the slope of the sigmoid. Different choices for activation functions may lead to variations in the specific dynamics, but the stability of the limit sets of interest would remain unchanged^30^.

A defining feature of epileptic seizures is persistent neuronal firing. When a large fraction of excitatory neurons become active, this heightened activity can sustain pathological firing through positive feedback. To model this mechanism, we introduce a term referred to as sustenance by replacing the original linear decay in Eqs. (1 and 2) with a second-order decay:

7 $$\dot{E}={\tau }_{E}\left({A}_{E}(1-E)-E\left(1-{q}_{E}E\right)\right)$$

8 $$\dot{I}={\tau }_{I}\left({A}_{I}(1-I)-I\left(1-{q}_{I}E\right)\right)$$

with dynamics restricted to *Ω*. The level of sustenance within the excitatory and inhibitory populations is quantified by the parameters *q*_*E*_ ∈ [0, 1] and *q*_*I*_ ∈ [0, 1], respectively. Equations (7 and 8) together represent the equations governing the interaction between one excitatory and one inhibitory neuronal population.

Accordingly, the excitatory nullcline is given by,

9 $${A}_{E}(1-E)-E(1-{q}_{E}E)=0$$

and the inhibitory nullcline is given by,

10 $${A}_{I}(1-I)-I(1-{q}_{I}E)=0.$$

### Modelling the depletion of inhibitory neurotransmitters

We model the depletion of inhibitory neurotransmitters as an activity-dependent process: the level of inhibitory neurotransmitter available at time *t* is influenced by the average inhibitory activity over a preceding time window Δ*t*. This depletion reduces the effective inhibition experienced by postsynaptic neurons, denoted as *I*^eff^, which we define as,

11 $${I}^{{\rm{eff}}}=I\left(1-\frac{\rho }{\Delta t}\mathop{\int}\nolimits_{\!\!t-\Delta t}^{t}I\left(\tau \right){\rm{d}}\tau \right),$$

where *ρ* ∈ [0, 1] is a constant that governs the level of depletion in inhibitory neurotransmitters.

Applying Taylor’s theorem to expand the integral in Eq. (11) about Δ*t* = 0 yields,

12 $$\mathop{\int}\nolimits_{\!\!t-\Delta t}^{t}I\left(\tau \right)d\tau =\left(\Delta t\right)I(t)-\frac{1}{2}{\left(\Delta t\right)}^{2}{\dot{F}}_{I}(t)+{\mathcal{O}}\left({\left(\Delta t\right)}^{3}\right).$$

Taking the first-order approximation of the above expansion and substituting into Eq. (11) yields,

13 $${I}^{{\rm{eff}}}\approx I\left(1-\rho I\right).$$

To incorporate inhibitory neurotransmitter depletion into our model, we replace *I* with *I*^eff^ in Eqs. (3 and 4), encoding the reduced inhibition felt by quiescent excitatory and inhibitory populations, while keeping the rest of the framework unchanged.

### Modelling depolarising GABAergic neurotransmission

Depolarizing GABAergic neurotransmission occurs when postsynaptic neurons accumulate chloride ions due to disrupted chloride homeostasis^35^. Two key factors contribute to this disruption: (1) dysfunction of chloride transporters and (2) activity-dependent chloride influx.

To model activity-dependent chloride influx, we note that excitatory activity depolarizes quiescent neurons, and when GABA_*A*_ receptors are activated, chloride influx follows. If chloride co-transporters fail to regulate this influx effectively, it can cause chloride accumulation^35^. We model this transporter imbalance using the parameter *κ*, where *κ* = 0 represents intact homeostasis and positive values indicate impairment, with higher values reflecting greater dysfunction.

Accordingly, we define the fraction of quiescent excitatory neurons with excessive chloride accumulation as: *p* = *κEI*. This expression captures the interplay between neuronal depolarization (*E*), GABA_*A*_ activation (*I*), and transporter dysfunction (*κ*) on promoting chloride accumulation.

With this definition, we subdivide the quiescent excitatory population into two subpopulations. The first subpopulation, comprising the fraction *p*, consists of neurons with high chloride accumulation, which perceive GABAergic input as excitatory^37^:

14 $${x}_{P}={a}_{EE}E+{a}_{PI}I+{D}_{E}$$

where *a*_*PI*_ represents the sensitivity of these neurons to the excitatory action of GABA. The second subpopulation, comprising the fraction 1 − *p*, consists of neurons for which GABAergic input remains inhibitory. Their perception of inputs remain identical to those of the standard excitatory population, as described by Eq. (3).

The dynamics of the excitatory population is derived by combining its two subpopulations, with the overall perception of the quiescent excitatory population represented as a weighted sum of the perceptions of the subpopulations:

15 $${x}_{R}=p{x}_{P}+(1-p){x}_{E}$$

To incorporate depolarising effect of GABA into our model, we replace *x*_*E*_ with *x*_*R*_ in the activation function governing the excitatory population (Eq. (5)), while keeping the rest of the framework unchanged.

### Modelling interventions

We model GABAergic enhancement by introducing a factor, *σ*_GABA_, to amplify the effect of inhibition in the argument of the excitatory activation function,

16 $${x}_{E}={a}_{EE}E-{\sigma }_{{\rm{GABA}}}{a}_{EI}I+{D}_{E}$$

Equation (16) is used in lieu of Eq. (3) in the excitatory activation function to simulate GABAergic enhancement, while the rest of the framework remains unchanged.

To model the suppression of rhythmic firing, we introduce a term *σ*_RS_ that counteracts sustenance by modifying Eqs. (7 and 8), replacing *q*_*E*_ and *q*_*I*_ with (*q*_*E*_ − *σ*_RS_) and (*q*_*I*_ − *σ*_RS_), respectively.

### Numerical simulation

All analyses were carried out in MATLAB. The nullclines were plotted using the function *fimplicit*, and the fixed points were obtained using the function *solve*. Limit cycles and other trajectories were simulated using the function *ode23s*. The bifurcation diagrams were generated through a numerical continuation analysis.

## Supplementary information

Supplementary Information

## Data Availability

This study used no experimental or observational data. All simulations were implemented using built-in MATLAB functions as described in the Methods section. The equations and parameter values provided in the manuscript are sufficient to reproduce the reported results. Example MATLAB scripts are available in the Github repository: https://github.com/Aero264/seizure_interventions.
