# Supplementary material for: Modelling dysfunction-specific interventions for seizure termination in epilepsy
Source: NPJ Syst Biol Appl. 2025 Dec 19;12:9. doi: 10.1038/s41540-025-00632-9 (PMC12800326; doi:10.1038/s41540-025-00632-9)
Supplement: Supplementary file 1 — Supplementary Information [file 41540_2025_632_MOESM1_ESM.pdf]

Modelling dysfunction-specific interventions for seizure termination in epilepsy

Aravind Kumar Kamaraj* and Matthew Parker Szuromi†

SUPPLEMENTARY MATERIAL

Supplementary Note 1. Seizures arising from the depletion of inhibitory neurotransmitter

In this section, we study seizures arising due to the depletion of inhibitory neurotransmitter by varying the depletion parameter ρ as described in Results section of the main text. Phase portraits depicting key stages of the evolution of neuronal activity as ρ increases are shown in Fig. S1. All other parameters are fixed at their baseline values.

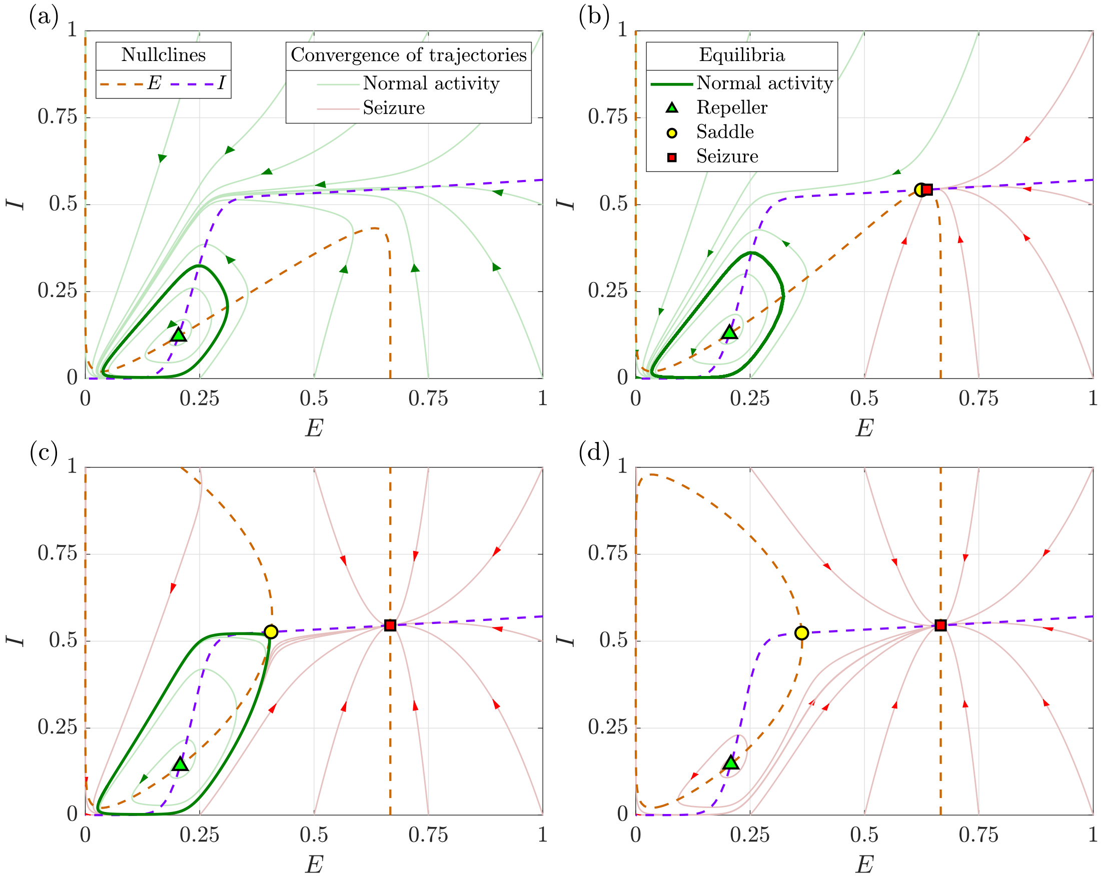

FIG. S1. Phase portraits illustrating the change in dynamics with an increase in the depletion parameter (ρ): (a) $\rho = 0$: normal activity is the sole attractor, (b) $\rho = 0.376$: birth of seizure attractor and saddle through a saddle-node bifurcation leading to bistability, (c) $\rho = 0.874$: normal activity vanishes through a saddle-homoclinic bifurcation, and (d) $\rho = 1$: seizure remains the sole attractor. All other parameters remain as in the baseline model.

Increasing ρ primarily affects the E-nullcline, shifting its local maximum away from the E-axis. At $\rho = 0$, the system exhibits a globally attracting limit cycle corresponding to normal activity as shown in Fig. S1(a). At $\rho \approx 0.3744$, E- and I-nullclines intersect again, giving rise to a saddle and the seizure attractor through a saddle-node bifurcation. Figure S1(b) depicts the phase portrait just after this bifurcation, illustrating bistability.

As ρ increases further, the limit cycle continues to expand until it eventually collides with the saddle and disappears through a saddle-homoclinic bifurcation at $\rho \approx 0.874$ as shown in Fig. S1(c). This destroys bistability, leaving seizure as the sole attractor: for $\rho > 0.874$, all trajectories converge onto the seizure attractor, as illustrated in Fig. S1(d).

* a.kamaraj@surrey.ac.uk
† mszuromi@bu.edu

Supplementary Note 2. Seizures arising from the depolarising effect of GABAergic neurotransmission

In this section, we study seizures arising due to the depolarising effect of GABAergic neurotransmission by varying the chloride accumulation parameter κ as described in Results section of the main text. Phase portraits depicting key stages of the evolution of neuronal activity as κ increases are shown in Fig. S2. The sensitivity parameter quantifying the effect of depolarising GABA on postsynaptic neurons, a_{PI}, is held constant at 5. All other parameters are fixed at their baseline values.

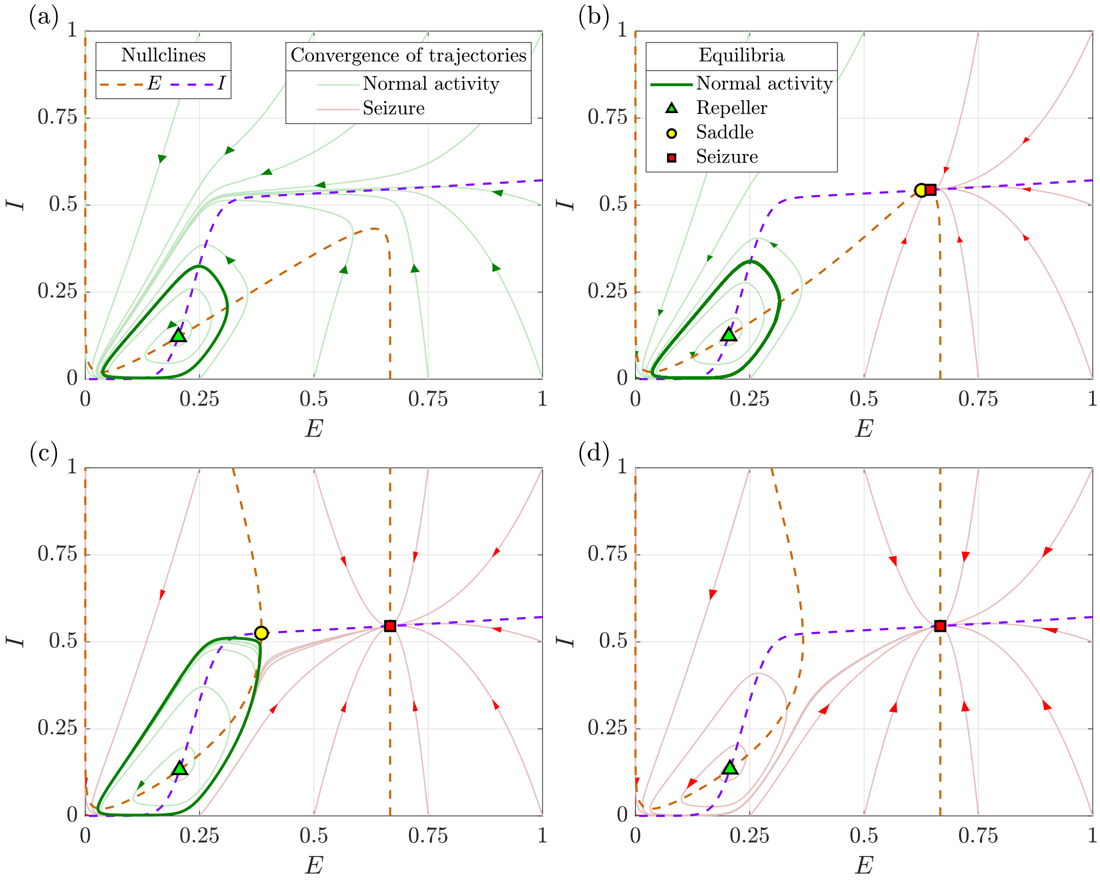

FIG. S2. Phase portraits illustrating the change in dynamics with an increase in the chloride accumulation parameter (κ): (a) $\kappa = 0$: normal activity is the sole attractor, (b) $\kappa = 0.4$: just after the birth of seizure attractor and saddle through a saddle-node bifurcation leading to bistability, (c) $\kappa \approx 1.61714$: normal activity vanishes through a saddle-homoclinic bifurcation, and (d) $\kappa = 1.8$: seizure remains the sole attractor. All other parameters remain as in the baseline model.

Increasing κ primarily affects the E-nullcline, similar to the case of inhibitory neurotransmitter depletion. At $\kappa = 0$, the system exhibits a globally attracting limit cycle corresponding to normal activity as shown in Fig. S2(a). At $\kappa \approx 0.394$, E- and I-nullclines intersect again, giving rise to a saddle and the seizure attractor through a saddle-node bifurcation. Figure S2(b) depicts the phase portrait just after this bifurcation, illustrating bistability.

As κ increases further, the limit cycle continues to expand until it eventually collides with the saddle and disappears through a saddle-homoclinic bifurcation at $\kappa \approx 1.61714$ as shown in Fig. S2(c). This destroys bistability, leaving seizure as the sole attractor: for $\kappa > 1.61714$, all trajectories converge onto the seizure attractor, as illustrated in Fig. S2(d).

Supplementary Note 3. GABAergic enhancement to counteract seizures arising from hyperexcitation

In this section, we study GABAergic enhancement (σ_{GABA}) as an intervention to terminate seizures arising from hyperexcitation ($D_E = 3$) as described in Results section of the main text. All other parameters are fixed at their baseline values. The corresponding bifurcation diagram is shown in Fig. S3. Phase portraits depicting key stages of the evolution of neuronal activity as σ_{GABA} increases are shown in Fig. S4.

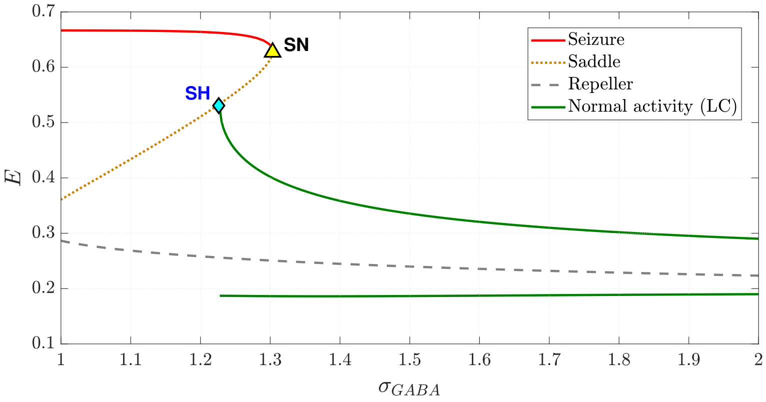

FIG. S3. Bifurcation diagram illustrating termination achieved by GABAergic enhancement (σ_{GABA}) in seizures arising from hyperexcitation ($D_E = 3$). The two solid green lines indicate the extrema of the limit cycle describing normal activity while the solid red line represents seizure. The saddle-node bifurcation is marked as 'SN' and the saddle-homoclinic bifurcation is marked as 'SH'.

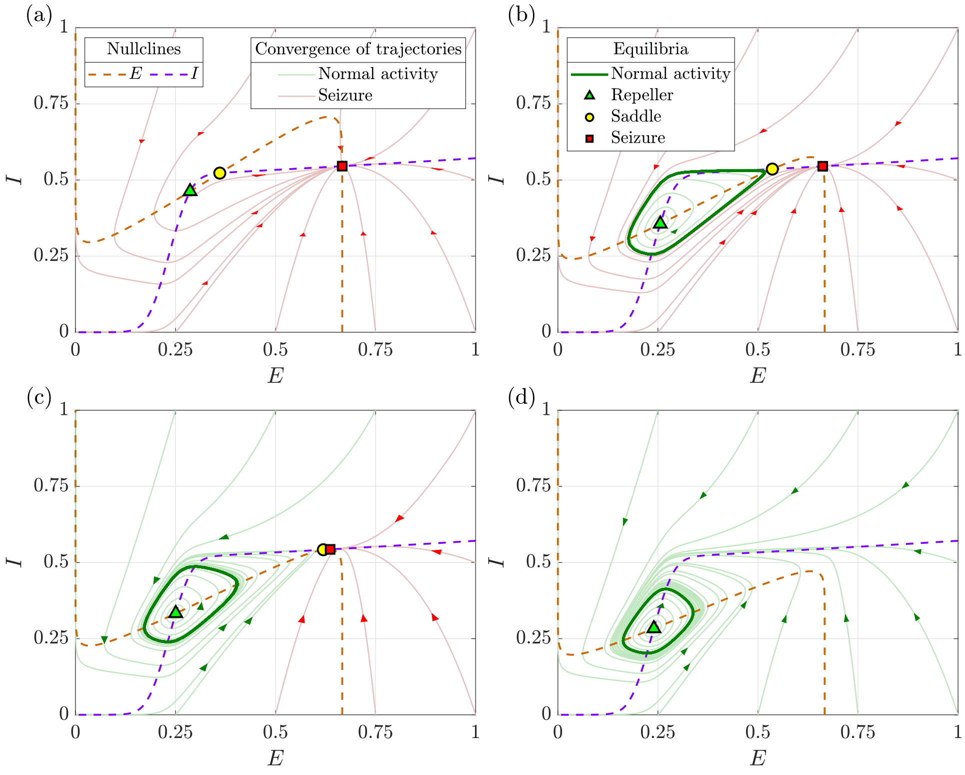

FIG. S4. Phase portraits illustrating termination achieved by GABAergic enhancement (σ_{GABA}) in seizures arising from hyperexcitation ($D_E = 3$): (a) $\sigma_{\mathrm{GABA}} = 1$: seizure is the sole attractor, (b) $\sigma_{\mathrm{GABA}} = 1.23$: birth of normal activity and saddle through a saddle-homoclinic bifurcation leading to bistability, (c) $\sigma_{\mathrm{GABA}} = 1.302$: just before seizure vanishes through a saddle-node bifurcation leading to termination, and (d) $\sigma_{\mathrm{GABA}} = 1.5$: normal activity remains the sole attractor. All other parameters remain as in the baseline model.

Supplementary Note 4. GABAergic enhancement to counteract seizures arising from the depletion of inhibitory neurotransmitter

In this section, we study GABAergic enhancement (σ_{GABA}) as an intervention to terminate seizures arising from the depletion of inhibitory neurotransmitter ($\rho = 1$) as described in Results section of the main text. All other parameters are fixed at their baseline values. The corresponding bifurcation diagram is shown in Fig. S5. Phase portraits depicting key stages of the evolution of neuronal activity as σ_{GABA} increases are shown in Fig. S6.

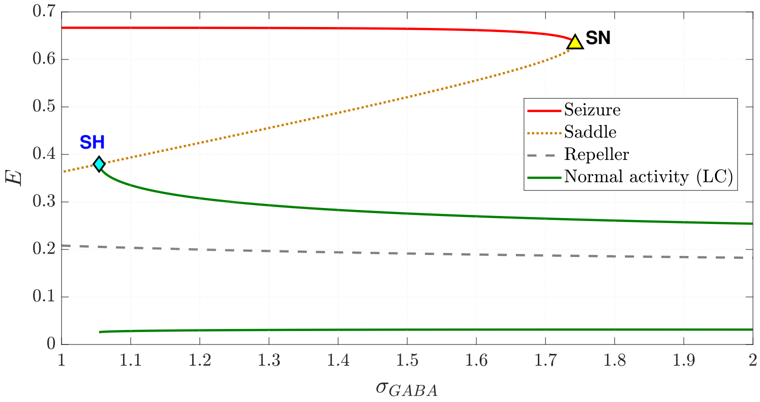

FIG. S5. Bifurcation diagram illustrating termination achieved by GABAergic enhancement (σ_{GABA}) in seizures arising from the depletion of inhibitory neurotransmitter ($\rho = 1$). The two solid green lines indicate the extrema of the limit cycle describing normal activity while the solid red line represents seizure. The saddle-node bifurcation is marked as 'SN' and the saddle-homoclinic bifurcation is marked as 'SH'.

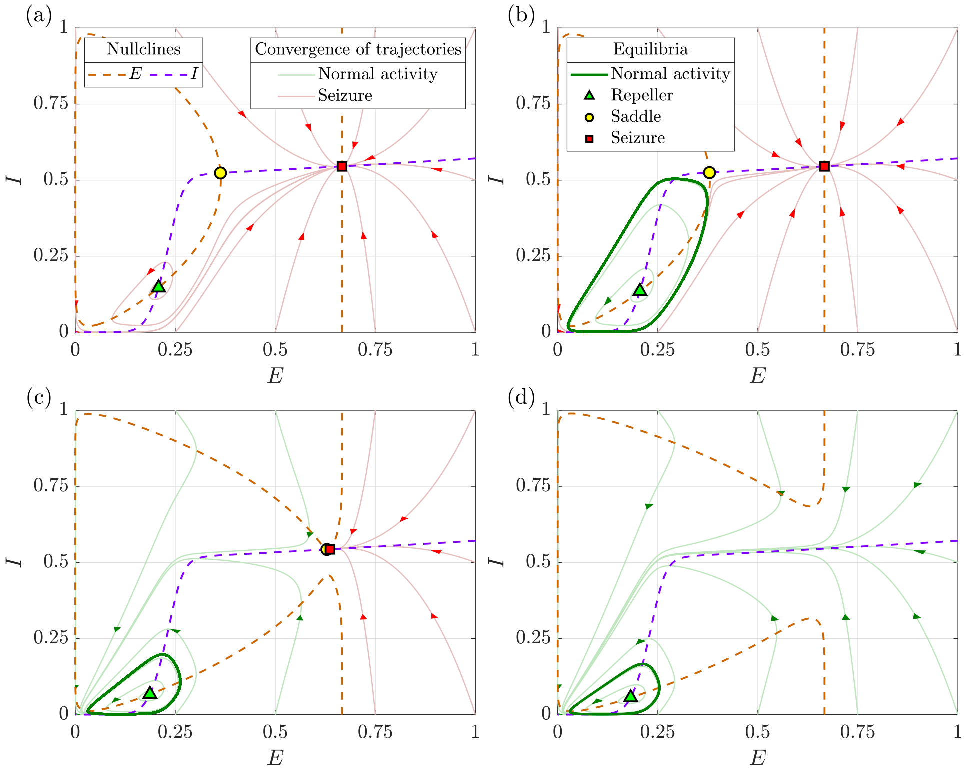

FIG. S6. Phase portraits illustrating termination achieved by GABAergic enhancement (σ_{GABA}) in seizures arising from the depletion of inhibitory neurotransmitter ($\rho = 1$): (a) $\sigma_{\mathrm{GABA}} = 1$: seizure is the sole attractor, (b) $\sigma_{\mathrm{GABA}} = 1.054$: birth of normal activity and saddle through a saddle-homoclinic bifurcation leading to bistability, (c) $\sigma_{\mathrm{GABA}} = 1.742$: just before seizure vanishes through a saddle-node bifurcation leading to termination, and (d) $\sigma_{\mathrm{GABA}} = 1$: normal activity remains the sole attractor. All other parameters remain as in the baseline model.

Supplementary Note 5. Rhythmic suppression to counteract seizures arising from the depolarising effect of GABAergic neurotransmission

In this section, we study rhythmic suppression ($\sigma_{\rm RS}$) as an intervention to terminate seizures arising from the depolarising effect of GABAergic neurotransmission ($\kappa = 1.8$ and $a_{PI} = 5$) as described in Results section of the main text. All other parameters are fixed at their baseline values. The corresponding bifurcation diagram is shown in Fig. S7. Phase portraits depicting key stages as $\sigma_{\rm RS}$ increases are shown in Fig. S8.

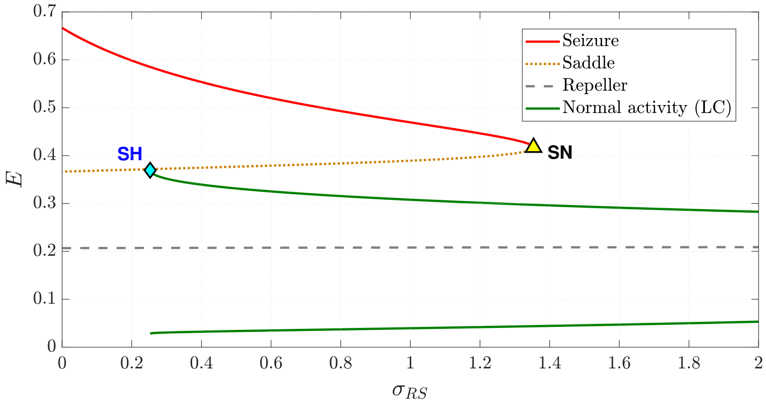

FIG. S7. Bifurcation diagram illustrating termination achieved by rhythmic suppression ($\sigma_{\rm RS}$) in seizures arising from the depolarising effect of GABAergic neurotransmission ($\kappa = 1.8$ and $a_{PI} = 5$). The two solid green lines indicate the extrema of the limit cycle describing normal activity while the solid red line represents seizure. The saddle-node bifurcation is marked as 'SN' and the saddle-homoclinic bifurcation is marked as 'SH'.

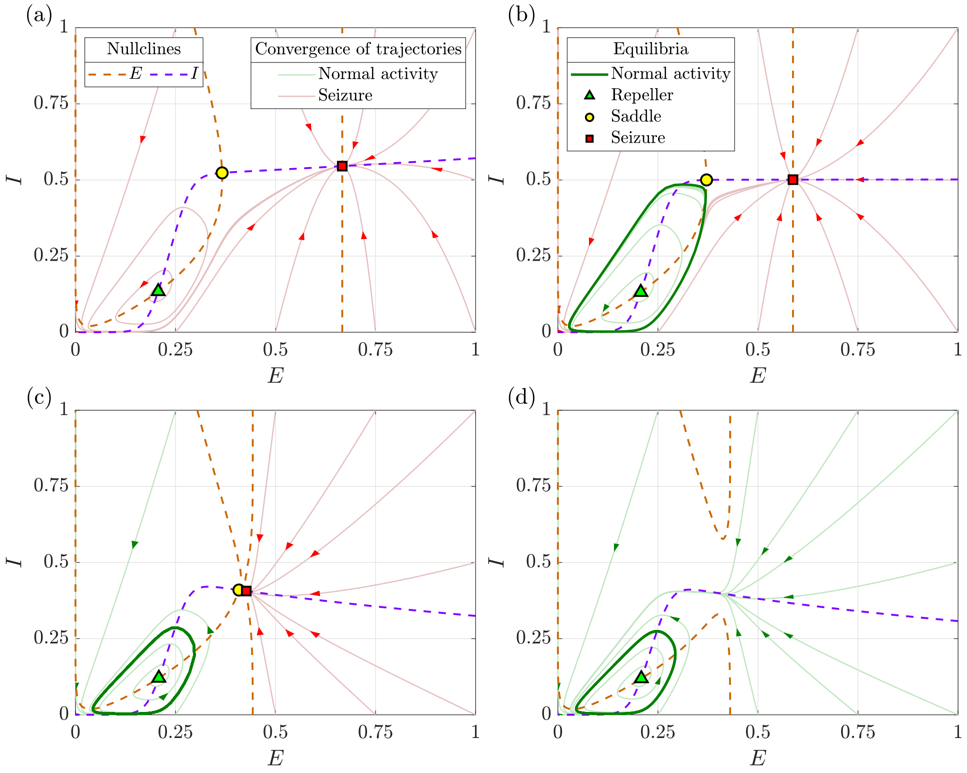

FIG. S8. Phase portraits illustrating termination achieved by rhythmic suppression ($\sigma_{\rm RS}$) in seizures arising from the depolarising effect of GABAergic neurotransmission ($\kappa = 1.8$ and $a_{PI} = 5$): (a) $\sigma_{\rm RS} = 0$: seizure is the sole attractor, (b) $\sigma_{\rm RS} = 0.243$: birth of normal activity and saddle through a saddle-homoclinic bifurcation leading to bistability, (c) $\sigma_{\rm RS} = 1.33$: just before seizure vanishes through a saddle-node bifurcation leading to termination, and (d) $\sigma_{\rm RS} = 1.5$: normal activity remains the sole attractor. All other parameters remain as in the baseline model.
